# Water permeability/impermeability in seeds of 15 species of *Caragana* (Fabaceae)

**DOI:** 10.7717/peerj.6870

**Published:** 2019-05-09

**Authors:** Dali Chen, Rui Zhang, Carol C. Baskin, Xiaowen Hu

**Affiliations:** 1 State Key Laboratory of Grassland Agro-ecosystems, Key Laboratory of Grassland Livestock Industry Innovation, Ministry of Agriculture and Rural Affairs, College of Pastoral Agriculture Science and Technology, Lanzhou University, Lanzhou, China; 2 Hainan Key Laboratory for Sustainable Utilization of Tropical Bioresources, College of Agriculture, Hainan University, Haikou, China; 3 Department of Biology, University of Kentucky, Lexington, KY, United States of America; 4 Department of Plant and Soil Sciences, University of Kentucky, Lexington, KY, United States of America

**Keywords:** Seed coat structure, Hardseededness, Imbibition, *Caragana*, Maternal environment, Seed storage

## Abstract

Majority legumes in the temperate and arctic zones have water-impermeable seeds (physical dormancy, PY). However, various authors have reported that seeds of some *Caragana* species are water-permeable and thus non-dormant. We (1) tested seeds of 15 species of *Caragana* matured in the same site in 2014, 2016 and/or 2017 for presence of PY, (2) determined if dry storage decreased or increased the percentage of seeds with PY and (3) located the site on the seed coat of 11 species where water enters the seed. Sixty-three percent and 45% of the seeds of *C. roborovskyi* had PY in 2016 and 2017, respectively, but only 0–14% of the seeds of the other 14 species had PY. The palisade layer in the seed coat of water impermeable seeds had no cracks in it, whereas cracks were present in the palisade layer of water-permeable seeds. Year of collection and dry storage had significant effects on imbibition of two species (*C. acanthophylla* and *C. roborovskyi*). In two (*C. acanthophylla* and *C. roborovskyi*) of the 11 species tested, the hilum was the site of water entry into seeds (control seeds, not any dormant broken treatments), but for the other nine species tested water entered through all parts of the seed coat.

## Introduction

The about 100 species of *Caragana* are mainly distributed from the arid areas of northern China to the humid and forested areas of East Asia; approximately 70 species occur in China, and differentiated into different species depending on the habitats (Zhang, 2005; Guan & Ma, 2014; Fang et al., 2017). *Caragana* species are a food resource for wild animals and are preferred for reforestation in regions where desertification and erosion are problems (Su et al., 2005; Fan, Freedman & Gao, 2007). In addition, their flowers are a good source of honey, and their seeds are used as an herbal medicine in China (Zhao et al., 2005).

Worldwide, seeds of the majority of Fabaceae species (legumes) in the temperate and arctic zones are water-impermeable, i.e., physical dormancy (PY) (Baskin & Baskin, 2014; Rubio de Casas et al., 2017). Previous studies have shown that seeds of *Caragana acanthophylla* (Wang et al., 2015), *C. boisi* (Fang et al., 2017), *C. roborovskyi* (Song et al., 2014), *C. sinica* (Li, Kang & Li, 2014) and *C. stipitata* (Fang et al., 2017) are water-impermeable, while those of *C. intermedia*, *C. licentiana* and *C. opulens* are water-permeable (no dormancy) (Zhao et al., 2005). Song et al. (2013) found that the percentage of permeable seeds of *C. roborovskyi* increased from 57% to 98% after 40 min of hot water treatment (70 °C) compared with the control. However, according to Fang et al. (2017), seeds of *C. roborovskyi* have no dormancy, with >90% germination. These controversial results may arise from differences among species, maternal environment (year of collection), degree of maturity when collected, and storage duration and conditions (Meisert, 2002; Van Assche & Vandelook, 2010; Li, Kang & Li, 2014; Jaganathan, 2016; Fang et al., 2017). For example, 5 years of dry storage at 20 °C resulted in an increase in percentage of seeds of three species (*Pelargonium australe*, *P. inquinans* and *P. zonale*) with impermeable seed coats (Meisert, 2002). However, Van Assche & Vandelook (2010) have reported that 88% of *Vicia sativa* seeds became water permeable after dry storage at room conditions (18–22 °C, 40% RH) for 12 months. Thus, determining the effect of species, collection year and seed storage on seed permeability to water would increase our knowledge of the occurrence PY in seeds of *Caragana*.

Generally, PY in Fabaceae is attributed to the presence of a water-impermeable palisade layer of cells in the seed coat (Baskin & Baskin, 2014). Fang et al. (2017) found that the palisade layer was more compact in two species of *Caragana* with low or no germination than it was in four species with high germination percentages, indicating that the palisade layer is responsible for seed coat impermeability. However, they did not investigate the site of water entry into seeds. Hu et al. (2008) reported that the primary site of water entry into sulfuric acid-treated seeds of *Sophora alopecuroides* (Fabaceae) was the hilum. However, in seeds of other Fabaceae, including those of *Gleditsia triacanthos* (Geneve, 2009), *Cladrastis kentukea* (Gama-Arachchige et al., 2013), *Derris scandens* (Jayasuriya et al., 2012) and *Robinia pseudoacacia* (Karaki et al., 2012), the lens is the primary site of water entry into the seed.

Previous studies have focused on the response of seed germination to environmental cues in *Caragana* (Zheng et al., 2004; Lai et al., 2016; Fang et al., 2017) and how to break PY of *Caragana* species (Song et al., 2013). Fang et al. (2017) tested the effect of temperature on seed germination and dormancy in 12 species of *Caragana* from several climatic regions of China. However, they did not consider maternal effects (year of collection) as a possible explanation for differences in dormancy among the 12 species. Thus, a comparative study of the seeds of *Caragana* species collected from the same site in different years would add to our understanding of seed coat permeability of *Caragana*. Our aims were to (1) determine the percentage of impermeable fresh and dry-stored seeds of *Caragana* species collected in different years; (2) compare the structure of the seed coat of *Caragana* species with and without PY; and (3) identify the site(s) of water entry into the seeds.

## Materials & Methods

### Seed collection

Mature seeds were collected from plants of 15 *Caragana* species growing in the Desert Botanical Garden in Minqin County (102°59′E, 38°34′N; 1,378 m a. s. l.), Gansu Province, China, in June and July in 2014, 2016 and 2017 (Table 1) (Seeds were used in our study were permited to collect by Changlong Li, who is the director of Minqin Deseart Botanical Garden). Unfortunately, not every species was collected in all years (Table 1). Pods were collected as soon as they were ripe, transported to the laboratory and dried for 1 or 2 d at room conditions (RH 35%, 20 °C). Seeds were manually separated from pods and then stored dry in a paper bag (gas exchange is free) at room conditions (RH 20–45%, 16–22 °C) until used. Seed moisture content (fresh seeds) of all species was determined by weighing seeds before and after drying at 130 °C for 2 h, seeds were ground before the moisture content was determined (Chinese National Standard, 2001) (Table S2). The thousand seed weight of 15 species were determined by weighing eight replicates of 100 seeds before experiments commenced.

**Table 1:** The 1,000-seed mass and year(s) of collection of the 15 *Cagagana* species at Minqin in Gansu, China.

Species	1,000-seed weight (g)	Year collected		
		2014	2016	2017
*C. acanthophylla*	14.02 ± 0.21	Y(*)	N	N
*C. arborescens*	24.68 ± 1.39	Y(*)	Y	Y
*C. bongardiana*	9.43 ± 0.08	Y(*)	Y	N
*C. erinacea*	8.12 ± 0.16	N	Y(*)	N
*C. intermedia*	28.13 ± 0.34	Y(*)	Y	Y
*C. korshinskii*	56.81 ± 0.70	Y(*)	Y	Y
*C. microphylla*	26.34 ± 0.41	Y(*)	N	Y
*C. microphylla var.cinarea*	28.28 ± 0.57	N	Y(*)	Y
*C. opulens*	13.71 ± 0.26	N	N	Y(*)
*C. pruinosa*	7.71 ± 0.10	N	Y(*)	N
*C. roborovskyi*	15.33 ± 0.30	N	Y(*)	Y
*C. rosea*	15.65 ± 0.28	Y(*)	Y	N
*C. spinosa*	10.95 ± 0.13	N	Y(*)	Y
*C. stenophylla*	10.66 ± 0.08	Y(*)	N	N
*C. zahlbruckneri*	11.87 ± 0.13	N	Y(*)	N

**Notes.**

Y yes N no

The 1,000-seed mass of all species was determined no more than 1 week after seed collection.

(*) year 1,000-seed weight was determined.

The garden where the seeds were collected was founded in 1974, and indigenous *Caragana* species as well as species from different places in China were established from seeds in the 1980s. Each year, the plants are flood-irrigated at the beginning of April, May, September, October and November, but they are not watered in June, July and August, i.e., during the period of seed maturation. The mean annual temperature for this site for the years 1990–2017 is 8.8 °C (the mean temperature during the period of seed moisture, June and July, from 1997 to 2017 is 22.6 °C), and average annual rainfall is 116 mm, most of which falls from July to September (meteorological station in Minqin).

### Imbibition

Permeability of fresh seeds collected in 2014, 2016 and 2017 was determined no more than 1 week after seed collection for each of the 15 species. Four replicates of 25 seeds were incubated in 11-cm-diameter Petri dishes on two sheets of filter paper (Shuangquan, Hangzhou, China) moistened with 10 ml distilled water. Seeds were incubated at 20 °C in light in a 12-hr photoperiod (white fluorescent light, photon irradiance of 60 µmol m-2 s-1 at 400–700 nm) for 14 d. The number of imbibed and non-imbibed seeds in each Petri dish was monitored daily for 14 d. A 14-d period was appropriate for seeds of all tested species, since there was no increase in number of imbibed seeds when the imbibition period was increased to 28 d. When a seed imbibed, there was a clear increase in size (twice or more larger than non-imbibed seed), thus imbibed and non-imbibed seeds could easily be distinguished visually. Length of the emerged radical of ≥2 mm was the criterion for germination. After 6 months of storage at room conditions, imbibition tests were conducted on seeds of the 15 species, as described above. After imbibition tests, all impermeable seeds were scarified with sand paper and incubated at 20 °C for 14 d. We found that all of the impermeable seeds were viable, and all of them germinated after they were made water-permeable.

### Point of water entry during imbibition

To determine the point of water entry into the seed, 40 seeds of each of 11 species were divided into four groups of 10 seeds each for treatments as follows: (1) no blockage applied (control); (2) blockage material (vaseline) applied to hilum area; (3) vaseline applied to lens area; and (4) vaseline applied to hilum + lens area. To determine if vaseline is an adequate blocking material, whole water-permeable seeds were completely covered with vaseline. None of the vaseline-covered seeds had imbibed after 14 days, indicating that vaseline completely inhibited water uptake.

After vaseline had been applied to seeds of each of the 11 species, seeds were placed in Petri dishes on two sheets of moist filter paper and incubated at 20 °C for 14 d as described above. Number of imbibed and of non-imbibed seeds in each dish was monitored daily. This experiment was performed in June 2017.

### Seed coat surface and structure features

Based on results of imbibition studies that seeds of *C.  acanthophyll*, *C. bongardiana*, *C. intermedia*, *C. pruinosa*, *C. roborovskyi*, *C. spinosa* and *C. stenophylla* non-imbibed and others did not, thus seeds of *C. acanthophylla*, *C. arborescens*, *C. korshinskii*, *C. microphylla* and *C. roborovskyi* were chosen for SEM studies of the surface and structure of the seed coat. Seeds of *C. acanthophylla* and *C. roborovskyi* were chosen for scanning if they were impermeable to water in the imbibition test described above. Seeds of the other three species were considered to be water-permeable based on data from the imbibition tests. Three seeds of each species were mounted on a stud, coated with gold and examined with a JSM-5600LV (JEOL, Japan) scanning electron microscope at 20 kV. To examine the palisade layer, intact seeds were cut along the direction of the hilum with a scalpel.

### Statistical analysis

The effects of species, year of collection and storage on imbibition were tested by fitting the data to generalized linear models (GLMs). Duncan’s multiple range test was used to compare means when significant differences were found. Independent t-tests were conducted to compare the means between fresh and stored seeds at the level of collection year. Seed imbibition was a probability ranging from 0 to 1, hence we applied a binomial estimation of the model to test the effects of blockage on seed imbibition. All data were processed with GenStat, version 18.0 (VSN International, Ltd., Hemel Hempstead, United Kingdom).

## Results

### Imbibition

Species, year of collection and storage had significant effects on imbibition of *Caragana* seeds. Up to 63% of *C. roborovskyi* seeds had PY, while only 0–14% of the seeds of the other 14 species had PY (Table 2). PY was present in 63 and 45% of fresh *C. roborovskyi* seeds in 2016 and 2017, respectively, and in 37 and 31%, respectively, after 6 months of dry storage. Fourteen and 5% of fresh and stored seeds of *C. acanthophylla* collected in 2014, respectively, had PY. Nine and 8% of fresh *C. spinosa* seeds collected in 2016 and 2017, respectively, had PY, but none of the seeds of this species for either year had PY after 6 months of dry storage. PY was present in 0–3% of both fresh and stored seeds of the other 12 species.

**Table 2:** Effect of species, collection year and storage on the percentage of water-impermeable seeds of 15 *Caragana* species. Permeability of fresh seeds collected in 2014, 2016 and 2017 was determined no more than 1 week after seed collection for each of the 15 species.

Species	2014		2016		2017	
	Fresh	Stored (6 months)	Fresh	Stored (6 months)	Fresh	Stored (6 months)
*C. acanthophylla*	14.0 ± 2.6^Aa^	5.0 ± 2.5^Ab^	–	–	–	–
*C. arborescens*	0.0 ± 0.0^B^	0.0 ± 0.0^B^	0.0 ± 0.0^C^	0.0 ± 0.0^B^	0.0 ± 0.0^C^	0.0 ± 0.0^B^
*C. bongardiana*	0.0 ± 0.0^B^	0.0 ± 0.0^B^	2.0 ± 1.2^C^	0.0 ± 0.0^B^	–	–
*C. erinacea*	–	–	0.0 ± 0.0^C^	0.0 ± 0.0^B^	–	–
*C. intermedia*	0.0 ± 0.0^B^	0.0 ± 0.0^B^	1.0 ± 1.0^C^	0.0 ± 0.0^B^	0.0 ± 0.0^C^	0.0 ± 0.0^B^
*C. korshinskii*	0.0 ± 0.0^B^	0.0 ± 0.0^B^	0.0 ± 0.0^C^	0.0 ± 0.0^B^	0.0 ± 0.0^C^	0.0 ± 0.0^B^
*C. microphylla*	0.0 ± 0.0^B^	0.0 ± 0.0^B^	–	–	0.0 ± 0.0^C^	0.0 ± 0.0^B^
*C. microphylla* var*.cinarea*	–	–	0.0 ± 0.0^C^	0.0 ± 0.0^B^	0.0 ± 0.0^C^	0.0 ± 0.0^B^
*C. opulens*	–	–	–	–	0.0 ± 0.0^C^	0.0 ± 0.0^B^
*C. pruinosa*	–	–	2.0 ± 1.2^C^	0.0 ± 0.0^B^	–	–
*C. roborovskyi*	–	–	63.0 ± 3.8^Aa^	37.0 ± 3.4^Abc^	45.0 ± 1.0^Ab^	31.0 ± 1.9^Ac^
*C. rosea*	0.0 ± 0.0^B^	0.0 ± 0.0^B^	0.0 ± 0.0^C^	0.0 ± 0.0^B^	–	–
*C. spinosa*	–	–	9.0 ± 1.0^Ba^	0.0 ± 0.0^Bb^	8.0 ± 4.6^Ba^	0.0 ± 0.0^Bb^
*C. stenophylla*	3.0 ± 1.9^B^	0.0 ± 0.0^B^	–	–	–	–
*C. zahlbruckneri*	–	–	0.0 ± 0.0^C^	0.0 ± 0.0^B^	–	–

**Notes.**

Different uppercase letters in the same column and different lowercase letters in the same row indicate significant difference at the 0.05 level. Duncan’s multiple range test was used to compare means when significant differences were found by ANOVA. Independent *t*-tests were conducted to compare the means between fresh and stored seeds at the level of collection year.

– no data

### Point of water entry during imbibition

The effect of blockage treatments on seed imbibition varied with the species (Table 3). Except for *C. acanthophylla* and *C. roborovskyi*, the number of imbibed seeds did not differ significantly among blockage treatments (Table 3). For seeds of *C. acanthophylla* and *C. roborovsky*, blockage of the hilum and of the hilum plus lens significantly reduced the number of imbibed seeds compared with control, while blockage of lens had no effect on seed imbibition (Table 3).

**Table 3:** Number of imbibed seeds of the 10 seeds tested for each of the 11 *Caragana* species after 14 days incubation in light at 20 °C.

Species	Harvest time	Control	Hilum	Lens	Hilum + Lens
*C. acanthophylla*	2014	10^a^	6^b^	8^ab^	5^b^
*C. arborescens*	2017	10	10	10	10
*C. bongardiana*	2016	10	10	9	8
*C. intermedia*	2014	10	10	10	10
*C. korshinskii*	2014	10	10	10	10
*C. microphylla*	2017	10	10	10	10
*C. opulens*	2017	10	10	10	10
*C. pruinosa*	2016	10	10	9	8
*C. roborovskyi*	2016	8^a^	3^b^	5^ab^	2^b^
*C. rosea*	2016	10	10	10	10
*C. stenophylla*	2014	10	10	10	10

**Notes.**

Values sharing a different lowercase letter in a row indicate significant differences at the 0.05 level, using a binomial estimation of the generalized linear models to test the effects of blockage on imbibition.

**Figure 1:**
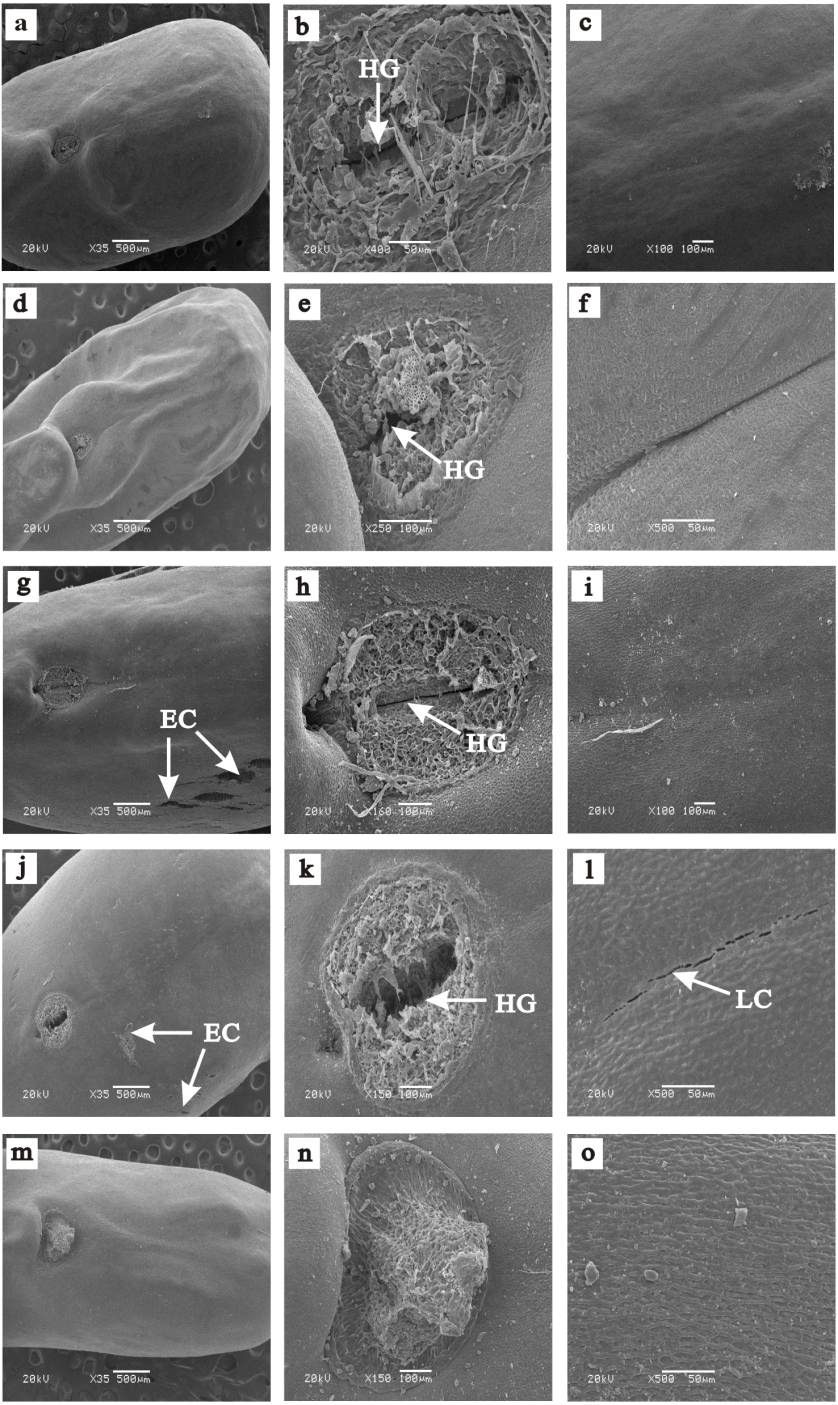
Scanning electron micrographs of the seed coat surface of five *Caragana* species. (A–C) *C. roborovskyi*: (A) intact seed; (B) hilum with opening in hilum groove (HG); (C) lens. (D–F) *C. acanthophylla*: (D) intact seed; (E) hilum with opening hilum groove (HG); (F) lens. (G–I) *C. korshinskii*: (G) intact seed and extrahilar region with cracks (EC); (H) hilum with opening in hilum groove (HG); (I) lens. (J–L) *C. arborescens*: (J) intact seed and extrahilar region with cracks (EC); (K) hilum with opening hilum groove(HG); (L) lens with cracks (LC). (M–O) *C. microphylla*: (M) entire seed; (N) hilum; (O) lens.

### Seed coat surface and structure features

**Figure 2:**
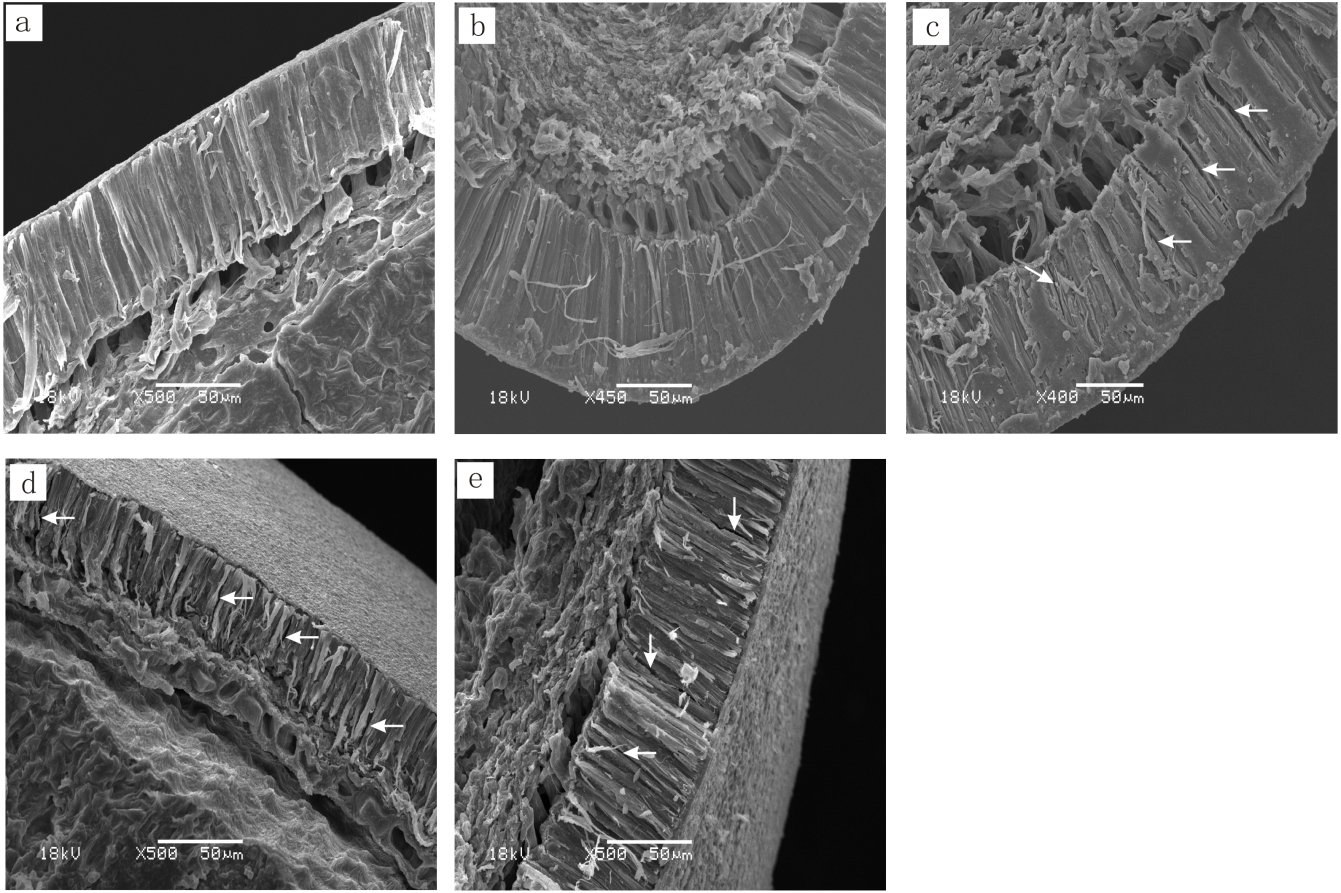
Scanning electron micrographs of the seed coat structure (intact seeds were cut along the direction of the hilum) structure of five *Caragana* species. (A) *C. roborovskyi*; (B) *C. acanthophylla*; (C) *C. korshinskii*; (D) *C. arborescens*; (E) *C. microphylla*. Arrows indicate that there was a crack in the palisade layer.

For all tested seeds, three distinct regions were present on the surface of the seed coat: (1) hilum with a groove in the middle (the micropyle lies on one side of the hilum and is covered by the hilum); (2) lens, which is on the opposite end of the hilum from the micropyle; and (3) extrahilar region, which includes the whole seed coat except the lens and hilum (Fig. 1). There was an obvious slit in the hilum of *C. acanthophylla* and *C. roborovskyi* seeds (Figs. 1B and 1E), and the lens and extrahilar region were intact and had no visible cracks (Figs. 1A, 1C, 1D and 1F). For seeds of *C. arborescens* and *C. korshinskii*, some obvious cracks were present in the hilum, lens and extrahilar region (Figs. 1G–1L). However, no visible cracks or silts were found on the seed surface of *C. microphylla* (Figs. 1M–1O). In addition, some obvious cracks were present in the palisade layer of *C. korshinskii*, *C. arborescens* and *C. microphylla* but not *C. acanthophylla* and *C. roborovskyi* seeds (Figs. 2C–2E).

## Discussion

### Seed imbibition

Physical dormancy is common in wild legumes, and the percentage of water-impermeable seeds varies with the species (Baskin & Baskin, 2014). In only two of 15 species of *Caragana* was PY ≥ 10%, i.e., *C. acanthophylla* and *C. roborovskyi*. This is consistent with previous studies of *Caragana* species in which only six of 23 *Caragana* species had >40% physically dormant seeds, suggesting that most seeds of most *Caragana* species do not have PY (Table S1).

Generally, PY is caused by a water-impermeable palisade layer of cells in the seed coat (Baskin & Baskin, 2014). Our results for five *Caragana* species indicated that the palisade layer was more compact in species with PY than in those without PY. Moreover, the palisade layer of those species without PY had some visible cracks in it, which would allow water to enter the seed. Among the 12 species tested, Fang et al. (2017) found that only seeds of *C. boisi* and *C. stipatata* were impermeable to water and that there were no cracks in the palisade layer of the seed coat of these two species. Thus, results from our study and those of Fang et al. (2017) on a total of 19 species of *Caragana* indicate that most *Caragana* species may lack a dense, water-impermeable palisade layer of cells.

Seed dormancy is affected by many factors, such as genetic basis, temperature, rainfall and radiation during seed development and maturation (Gresta, Avola & Abbate, 2007; Baskin & Baskin, 2014; Fang et al., 2017). Previous studies (Zhao et al., 2005; Song et al., 2013; Li, Kang & Li, 2014; Wang et al., 2015; Fang et al., 2017) have found that the difference in permeability of seeds among species may be due to environment conditions, genetic background and their interactions. In our study, the mother plants of testing seeds were grown in a common garden, thus this difference is caused by genetic background. Moreover, it is worth noting that data for 2016 and 2017 indicate that 63% and 45% of the fresh seeds of *C. roborovskyi* were impermeable to water, respectively. Thus results from our study indicate that genetics have a vital impact on physical dormancy of *Caragna* species.

Moreover, PY is affected not only by genetic background but also by the maternal environment during seed development (D’hondt, Brys & Hoffmann, 2010; Tozer & Ooi, 2014). In general, seeds matured under dry, high temperature conditions are more likely to form an impermeable seed coat than those produced under moist, low temperature conditions (Baskin & Baskin, 2014; Jaganathan, 2016). In our study, 63% and 45% of the fresh seeds of *C. roborovskyi* were impermeable in 2016 and 2017, respectively. Rainfall during the period of seed maturation (June and July) in 2016 (33.8 mm) was lower than that in 2017 (41.1 mm) (Fig. S1), and thus this difference may be related to the higher percentage of impermeable seeds of *C. roborovskyi* in 2016. However, differences in the temperature during the seed maturation period of these two years were minor (24.5 °C in 2016 and 24.3 °C in 2017). Moreover, there was no effect of year of collection in the other eight species for which seeds were collected in two or three years. For these eight species, impermeability was ≤ 9% and in most cases 0% (Table 2).

In some legumes, part (or all) of the seeds with PY stored dry at room temperatures for several months (or years) become permeable (Norton et al., 2002; Gresta, Avola & Abbate, 2007; Galindez et al., 2010). In our study, the percentage of impermeable seeds of *C. roborovskyi* and *C. spinosa* significantly decreased after dry storage at room conditions for 6 months, regardless of harvest year. Our results are similar to those of Wang et al. (2015), who found that the germination percentage of *C. acanthophylla* increased to varying degrees during dry storage at room conditions for 1, 6 and 8 years. In summary, our results showed that maternal environment and storage have an effect on PY. Therefore, maternal environment and storage should be considered when evaluating the proportion of seeds that has PY.

### Point of water entry during imbibition

Dormant seeds (after breaking PY) of legume species absorb water mostly via the lens (Gama-Arachchige et al., 2013; Baskin & Baskin, 2014). However, in our study, only blockage of the hilum of *C. acanthophylla* and *C. roborovskyi* significantly reduced the percentage of imbibed seeds. Thus, the hilum may be the site of water entry into seeds of *C. acanthophylla* and *C. roborovskyi*. This conclusion is supported by SEM results, which indicated that there are visible cracks only in the hilum of seeds of these two species. Hu et al. (2008) found that seeds of *Sophora alopecuroides* (Fabaceae) absorb water only via the hilum. Further, Gama-Arachchige et al. (2013) reported that the pseudolens is the site of water entry in seeds of *Cercis canadensis* (Fabaceae) and *Bauhinia acuminata* (Fabaceae).

For those *Caragana* species in which ≤10% of the seeds had PY, there was no significant difference in permeable seeds based on the different blocking treatments, and some obvious cracks were present in most regions (including hilum, lens and extrahilar region) of the seeds, suggesting that seeds of these species absorb water throughout the seed surface. Taylor (2004) reported that water entered seeds of *Ornithopus compressus* (Fabaceae) through some minute openings in the hilum and extrahilar region. Ma et al. (2004) suggested that fluorescent dyes could enter through some minute cracks in the cuticle of the seed coat penetrated into seeds of soybean. However, we did not find any cracks in seed coats of *C. microphylla*, although seeds of this species have no dormancy.

## Conclusions

In summary, percentage of *Caragana* seeds with PY varies with species, and most or all seeds of most species do not have PY. The percentage of seeds with PY may vary between years due to maternal effects. This is the first report of the site of water entry into seeds of *Caragana.* The hilum was the site of water entry into dormant seeds of *C. acanthophylla* and *C. roborovskyi,* and species without PY absorbed water via all parts of the seed coat.

##  Supplemental Information

10.7717/peerj.6870/supp-1 Figure S1 Mean monthly rainfall and mean monthly daily temperature at the seed collection site in 2014, 2016 and 2017 Each data point indicates mean monthly rainfall and temperature.

10.7717/peerj.6870/supp-2 Table S1 Permeability to water of seeds of 23 *Caragana* species **Y, yes** (the percentage of impermeable seeds < 10%)**; N, no** (percentage of impermeable seeds > 40%).

10.7717/peerj.6870/supp-3 Table S2 The moisture content of fresh seeds of the 15 *Cagagana* species at Minqin in Gansu, China Seed moisture content (fresh seeds) of all species was determined no more than 1 week after seed collection. –no data.

10.7717/peerj.6870/supp-4 Dataset S1 The 1,000-seed mass of seeds of the 15 *Cagagana* species Each data point indicates the 100-seed mass of seeds of *Cagagana* species.

10.7717/peerj.6870/supp-5 Dataset S2 The percentage of water-impermeable seeds of 15 *Caragana* species Each data point indicates impermeable seeds, –means no data.

10.7717/peerj.6870/supp-6 Dataset S3 Mean monthly rainfall and mean monthly daily temperature at the seed collection site Each data point indicates mean monthly rainfall and mean monthly daily temperature, long-term means from 1990 to 2017.

10.7717/peerj.6870/supp-7 Dataset S4 The moisture content of fresh seeds of the 15 *Cagagana* species Each data point indicates seed moisture content, –means no data.

## Additional Information and Declarations

### Field Study Permissions

The following information was supplied relating to field study approvals (i.e., approving body and any reference numbers): Collection of sees were used in our study were permited by the Minqin Deseart Botanical Garden.

### Data Availability

The following information was supplied regarding data availability: The raw data are available in Datasets S1–S4.
