# Supplementary figures and images for: Water permeability/impermeability in seeds of 15 species of Caragana (Fabaceae)
Source: PeerJ. 2019 May 9;7:e6870. doi: 10.7717/peerj.6870 (PMC6511390; doi:10.7717/peerj.6870)

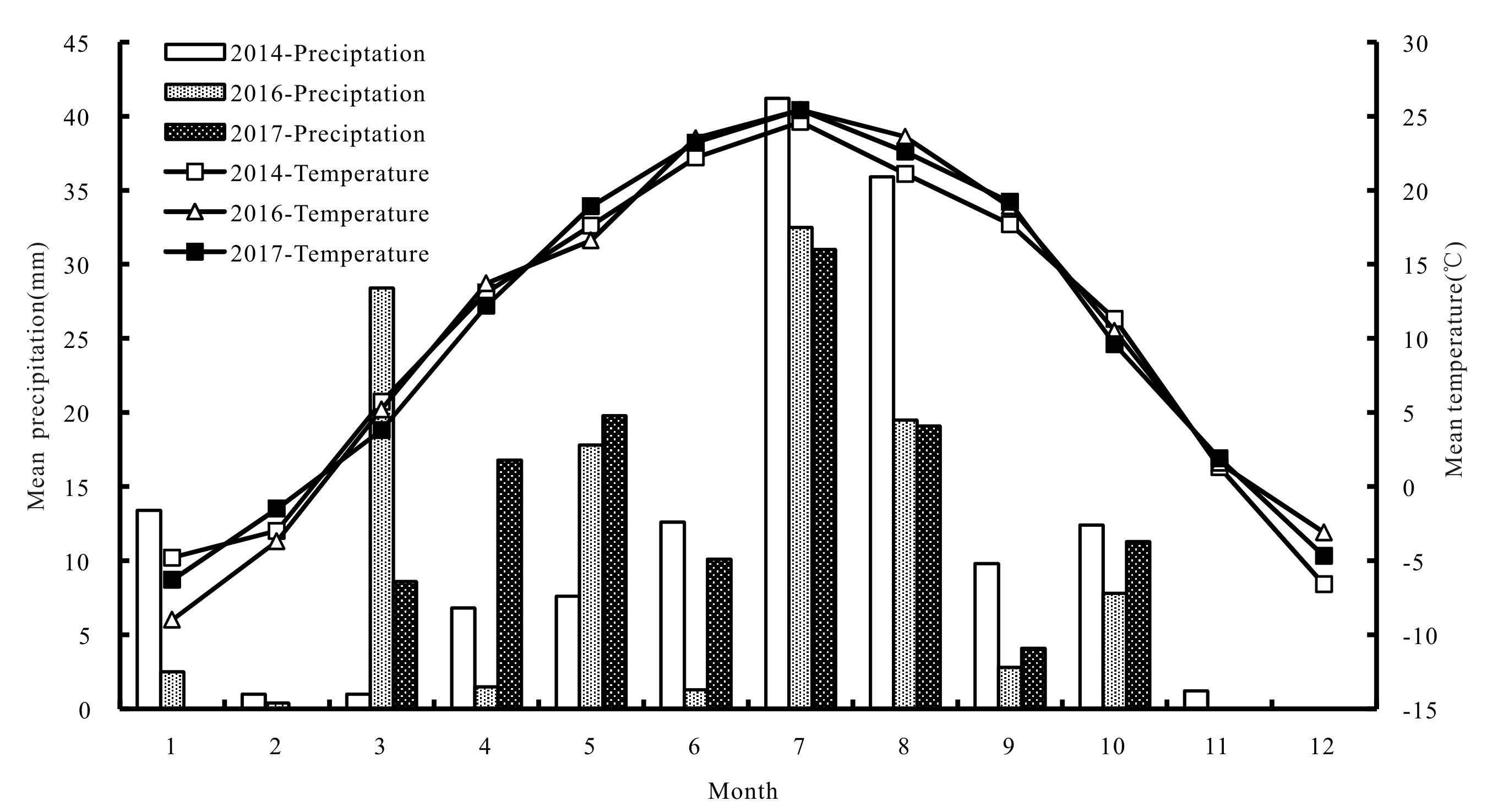

Supplement: Figure S1 — Each data point indicates mean monthly rainfall and temperature. [file peerj-07-6870-s001.png]
