# Supplementary material for: Water permeability/impermeability in seeds of 15 species of Caragana (Fabaceae)
Source: PeerJ. 2019 May 9;7:e6870. doi: 10.7717/peerj.6870 (PMC6511390; doi:10.7717/peerj.6870)
Supplement: Table S1 — Y, yes (the percentage of impermeable seeds < 10%); N, no (percentage of impermeable seeds > 40%). [file peerj-07-6870-s002.doc]

**TABLE S1 Permeability to water of seeds of 23 *Caragana* species.**

**Y, yes** (the percentage of impermeable seeds < 10%)**; N, no** (percentage of impermeable seeds > 40%)

| Species | Seed permeable to water | References |
| --- | --- | --- |
| *C. acanthophylla* | N | Li *et al*., 2009; Song *et al*., 2013; Wang *et al*., 2015 |
| *C. arborescens* | Y | Dietz *et al*., 2008; Sun *et* *al*., 2012; Fang *et al*., 2017 |
| *C. aurantiaca* | Y | Miao *et al*., 2009 |
| *C. boisi* | N | Zhao *et al*., 2015; Wu, 2016; Fang *et al*., 2017 |
| *C. brevifolia* | Y | Xu, 2007; Cui, 2008 |
| *C. bongardiana* | Y | Sun *et al*., 2012 |
| *C. erinacea* | Y | Sun *et al*., 2012 |
| *C. fruticosa* | Y | Yang *et al*., 2012 |
| *C. intermedia* | Y | Cai *et al*., 2011; Sun *et al*., 2012; Abudureheman *et al*., 2014; Lai *et al*., 2016; Mi *et al*., 2015; Fang *et al*., 2017; |
| *C. jubata* | N | Wang *et al*., 2009 |
| *C. korshinskii* | Y | Liu *et al*., 2011; Sun *et al*., 2012; Abudureheman *et al*., 2014; Lai *et al*., 2016; Yan *et al*., 2016; Fang *et al*., 2017; |
| *C. leucophloea* | Y | Li *et al*., 2009; Miao *et a*l., 2009; Fang *et al*., 2017 |
| *C. licentiana* | Y | Zhao *et al*., 2004; Zhao *et al*., 2005 |
| *C.microphylla* | Y | Zhang *et al*., 2011; Zhu *et al*., 2013; Abudureheman *et al*., 2014; Lai *et al*., 2016; Fang *et al*., 2017 |
| *C. opulens* | Y | Zhao *et al*., 2004; Zhao *et al*., 2005; Fang *et al*., 2017 |
| *C. pumila* | Y | Li *et al*., 2009; Song *et al*., 2011 |
| *C. roborovskyi* | N | Song *et al*., 2013 |
| *C. rosea* | Y | Wen *et al*., 2016; Fang *et al*., 2017 |
| *C. sinica* | N | Li *et al.*, 2013; Li *et a*l., 2014 |
| *C. stenophylla* | Y | Dong *et al*., 2015; Luo *et al*., 2015; Fang *et al*., 2017 |
| *C. stipitata* | N | Zhao *et al*., 2015; Fang *et al*., 2017 |
| *C. tibetica* | Y | Li *et al*., 2014; Fang *et al*., 2017 |
| *C. versicolor* | Y | Wang *et al*., 2009 |
