# Supplementary material for: Water permeability/impermeability in seeds of 15 species of Caragana (Fabaceae)
Source: PeerJ. 2019 May 9;7:e6870. doi: 10.7717/peerj.6870 (PMC6511390; doi:10.7717/peerj.6870)
Supplement: Table S2 — Seed moisture content (fresh seeds) of all species was determined no more than 1 week after seed collection. –no data. [file peerj-07-6870-s003.doc]

**TABLE S2 The moisture content of fresh seeds of the 15 *Cagagana* species at Minqin in Gansu, China**.

Seed moisture content (fresh seeds) of all species was determined no more than 1 week after seed collection.

-- no data.

| Species | seed moisture content | | |
| --- | --- | --- | --- |
|  | 2014 | 2016 | 2017 |
| *C. acanthophylla* | 6.40 ± 0.18 | -- | -- |
| *C. arborescens* | 6.97 ± 0.10 | 6.26 ± 0.08 | 6.71 ± 0.0 |
| *C. bongardiana* | 7.39 ± 0.09 | 7.24 ± 0.05 | -- |
| *C. erinacea* | -- | 6.73 ± 0.09 | -- |
| *C. intermedia* | 7.05 ± 0.08 | 6.39 ± 0.08 | 6.74 ± 0.09 |
| *C. korshinskii* | 7.48 ± 0.14 | 6.75 ± 0.07 | 6.75 ± 0.15 |
| *C. microphylla* | 6.42 ± 0.13 | -- | 6.25 ± 0.08 |
| *C. microphylla var.cinarea* | -- | 6.45 ± 0.05 | 6.67 ± 0.07 |
| *C. opulens* | -- | -- | 7.30 ± 0.11 |
| *C. pruinosa* | -- | 7.23 ± 0.10 | -- |
| *C. roborovskyi* | -- | 5.85 ± 0.09 | 6.29 ± 0.10 |
| *C. rosea* | 7.76 ± 0.07 | 7.17 ± 0.10 | -- |
| *C. spinosa* | -- | 7.00 ± 0.08 | 7.24 ± 0.09 |
| *C. stenophylla* | 6.85 ± 0.07 | -- | -- |
| *C. zahlbruckneri* | -- | 7.20 ± 0.10 | -- |
